# Grossesse et accouchement des patientes ayant un antécédent de césarienne à Dakar: aspects épidémio-cliniques thérapeutiques et pronostiques

**DOI:** 10.11604/pamj.2017.27.135.11924

**Published:** 2017-06-22

**Authors:** Cyr Espérance Koulimaya-Gombet, Abdoul Aziz Diouf, Moussa Diallo, Anna Dia, Codou Sène, Jean Charles Moreau, Alassane Diouf

**Affiliations:** 1 Centre Hospitalier National de Pikine, Service de Gynécologie et d’Obstétrique, Dakar, Sénégal; 2 Clinique Gynécologique et Obstétricale de l’Hôpital Aristide Le Dantec, Dakar, Sénégal

**Keywords:** Grossesse, utérus cicatriciel, accouchement, Sénégal, Pregnancy, trial of scar, delivery, Senegal

## Abstract

Il s’agissait de déterminer la fréquence hospitalière de l’accouchement sur utérus cicatriciel à Pikine, d’évaluer la qualité de la prise en charge anténatale des grossesses sur utérus cicatriciel et de rechercher les facteurs pronostiques de l’issue d’une épreuve utérine. Nous avons procédé à une étude rétrospective à partir de dossiers médicaux et des protocoles opératoires des patientes ayant accouché sur utérus cicatriciel durant la période du 1er Janvier 2010 au 31 Décembre 2011. Ont été analysés les données socio-démographiques, le suivi de la grossesse, les modalités thérapeutiques et le pronostic. Les données étaient saisies et analysées à l’aide du logiciel Microsoft Office Excel 2007 et du logiciel SPSS version 17.0. La fréquence des accouchements sur utérus cicatriciel était de 9,6%. L’âge moyen des patientes était de 29,4 ans. Les primipares représentaient 54%. Un espace inter-génésique court était retrouvé dans 52,6% des cas. En fonction du nombre de césarienne, la répartition était la suivante: 79,8% des cas d’utérus unicicatriciels, 17,9% pour les bicicatriciels et 2,3% pour les tricicatriciels. Le nombre de consultations prénatales effectuées était supérieur ou égal à 3 dans 79,8% des cas. Les patientes évacuées représentaient 54,2% et elles étaient déjà en travail à l’admission dans 81,7% des cas. Une épreuve utérine était autorisée chez 177 patientes (34,3%), et à l’issue de cette épreuve, 147 patientes avaient accouché par voie basse, soit 83% dont 21,7% par ventouse obstétricale. L’accouchement par césarienne était réalisé dans 71,4% des cas avec 245 césariennes en urgence et 93 césariennes programmées. Un antécédent d’accouchement par voie basse était un facteur déterminant d’un accouchement normal (p = 0,0001). Il existait également un lien significatif entre le mode d’admission et la décision de césarienne (p = 0,0001). La mortalité maternelle était de 0,4%. La mortalité périnatale était de 28,2‰ naissances vivantes. Nous assistons à une augmentation fulgurante des accouchements sur utérus cicatriciel dans nos centres de SONUC. La qualité de la prise en charge doit être rehaussée par une meilleure stratégie de préparation à l’accouchement. L’épreuve utérine est une procédure à encourager pour réduire le taux de césarienne itérative.

## Abstract (en)

The aim of our study was to determine hospitalization rate for vaginal birth after cesarean section in Pikine, to evaluate the quality of the management of pregnant women with previous cesarean section and to determine prognostic factors of the outcome of a trial of scar. We conducted a retrospective study based on medical records and operational protocols of patients who underwent vaginal birth after cesarean section over the period 1 January 2010 - 31 December 2011. We analyzed socio-demographic data, pregnancy follow-up, therapeutic modalities and prognosis. Data were collected and analyzed using Microsoft Office Excel 2007 software and SPSS software 17.0. The frequency of vaginal births after cesarean section was 9.6%. The average age of our patients was 29.4 years. Primiparous women accounted for 54%. Short spacing interval between births was found in 52.6% of cases. Based on the number of cesarean sections, the breakdown was as follows: patients with a history of one previous cesarean section (79.8%), patients with a history of two previous caesarean sections (17.9%) and patients with a history of three previous caesarean sections (2.3%). The number of antenatal consultations performed was greater than or equal to 3 in 79.8% of cases. Patients undergoing evacuation accounted for 54.2% and they were already in labor at the time of admission in 81.7% of cases. Trial of scar was authorized in 177 patients (34.3%) and, at the end of this test, 147 patients (83%) had vaginal birth, of whom 21.7% by vacuum extraction. Cesarean section was performed in 71.4% of cases with 245 emergency cesarean sections and 93 scheduled cesarean sections. A history of vaginal birth was a determining factor in normal delivery (p = 0.0001). There was also a significant relationship between mode of admission and decision to perform a cesarean section (p = 0.0001). Maternal mortality was 0.4%. Perinatal mortality rate was 28.2‰ of live births. We are witnessing a dramatic increase of deliveries after cesarean section in our SONUC Health Centre. The quality of management should be enhanced through a better strategy in preparation for delivery. Trial of scar is a procedure to encourage in order to reduce the rate of iterative cesarean sections.

## Introduction

La pratique de la césarienne n’a cessé d’augmenter au cours des deux dernières décennies dans la plupart des pays du monde. La césarienne itérative par crainte des risques maternels et périnatals est reconnue comme une des principales causes de cette inflation. Une césarienne sur trois est réalisée du fait d’un utérus cicatriciel [1]. En 1988, l’American Collège of obstetric and Gynecology (ACOG) soutenait pour la première fois l’option de la tentative de l’épreuve sous réserve [2]. Les recommandations actuelles sont en faveur d’une tentative de l’accouchement par voie basse en cas de cicatrice unique et en l’absence d’une indication médicale permanente [1, 3, 4]. Les taux de succès rapportés de la tentative d’accouchement par voie basse varient d’une région à l’autre. Ils étaient de 25% aux Etats Unis en 1993 [5], de 50% en Europe en 1994 [6] et entre 1,5% et 3,7% en Afrique subsaharienne dans les années 1990 [3]. A Dakar, entre 2004 et 2011, le taux de césariennes itératives est passé de 7,5% à 12,5% [7]. Il nous semblait donc important de faire le point sur cette question au Centre Hospitalier National (CHN) de Pikine, qui est actuellement l’un des plus importants centres de référence obstétricale à Dakar. Le but de ce travail était d’apporter notre contribution à l’amélioration de la prise en charge des accouchements en cas d’utérus cicatriciel. Ses objectifs étaient de déterminer la fréquence de l’accouchement sur utérus cicatriciel au CHN de Pikine, d’évaluer la qualité de la prise en charge des grossesses sur utérus cicatriciel, et de rechercher les facteurs pronostiques de l’issue d’une épreuve utérine.

## Méthodes

Cette étude s’est déroulée dans le service de Gynécologie et d’Obstétrique du Centre Hospitalier National (CHN) de Pikine à Dakar au Sénégal qui enregistre en moyenne 2700 accouchements par an. Il s’agissait d’une étude rétrospective descriptive portant sur les accouchements sur utérus cicatriciels durant la période du 1er Janvier 2010 au 31 Décembre 2011 (24 mois). Etait incluse toute patiente porteuse d’une grossesse avec un antécédent de césarienne et ayant accouché dans le service. Les cicatrices d’intervention gynécologique étaient les seuls critères de non inclusion (rupture utérines spontanée, perforation lors d’un curetage, myomectomie hysteroplastie, perforation et résections dans les salpingectomies). La collecte des données était possible à partir des dossiers médicaux et des comptes rendus opératoires. Pour la mère, le nombre et les indications des césariennes antérieures, l’espace inter-génésique, le lieu des consultations prénatales, l’existence d’accouchement antérieur par voie basse, le mode d’admission, les modalités de l’accouchement de la grossesse actuelle, les indications d’une éventuelle césarienne actuelle et les complications étaient enregistrés. Pour le nouveau-né, étaient pris en compte le score d’Apgar (à la 1^ère^ et à la 5^ème^ minute) et le poids de naissance. Le test de student était utilisé pour la comparaison des moyennes. Le seuil de significativité était fixé à 0,05.

## Résultats

Au cours de la période d’étude, sur 5341 accouchements, 515 patientes étaient porteuses d’un utérus cicatriciel, représentant un taux de 9,6%. L’âge moyen des patientes était de 29,4 ans (extrêmes de 18 et 47 ans). La parité moyenne était de 1,8 avec des extrêmes de 1 et 10. Les primipares étaient majoritaires (54,2%). Un espace inter-génésique compris entre un et 2 ans était retrouvé dans 51% des cas; 8 patientes avaient contracté une grossesse dans un délai inférieur à 12 mois après l’accouchement par césarienne (Tableau 1). Les utérus uni-cicatriciels (cicatrice unique) étaient beaucoup plus rencontrés dans notre étude avec 79,8% des cas, les bicicatriciels dans 17,9% des cas, et les utérus tricicatriciels ne représentaient que 2,3% des cas. Parmi les pathologies associées à la grossesse, l’hypertension artérielle et ses complications étaient les plus rencontrées (92,2%). Par ailleurs une anémie et un fibrome prævia étaient retrouvés respectivement dans 3,9% et 2,9% des cas. Les patientes avaient bénéficié d’une césarienne dans 71,4% des cas dont 47,6% en urgence.

**Table:** Répartition des patientes selon l’espace intergénésique

Durée en année	Nombre de cas	Pourcentage (%)
≥ 2	195	37,9
1-2	263	51
< 1	8	1,6
Non précisé	49	9,5
Total	515	100

Pour le reste (177 cas), une épreuve utérine était réalisée. A l’issue de cette épreuve, 147 patientes avaient accouché par voie basse; les 30 autres patientes (5,8%) avaient finalement accouché par césarienne après échec de l’épreuve utérine (Tableau 2). Un accouchement antérieur par voie basse était significativement associé à la réussite de l’épreuve utérine (p = 0,0001). Il existait également un lien statistiquement significatif (Tableau 3) entre le mode d’admission et la voie d’accouchement (p = 0,0001). Celles qui étaient venues d’elles-mêmes avaient subi une césarienne dans 70% des cas. Toutes les patientes référées avaient accouché par voie haute.

**Table:** Répartition des patientes selon le mode d’accouchement

Mode d’accouchement	Nombre	Pourcentage (%)
Césarienne	368	71,4
Programmée	93	18
Après épreuve utérine	30	5,8
En urgence	245	47,6
Voie basse	147	28,6
Total	515	100

**Table:** Antécédents d’accouchement par voie basse, mode d’admission etvoie d’accouchement

		Voie d’accouchement			
		Césarienne	Voie basse	Total	p
Accouchement précédent par voie basse	Non	278	89	367	0,0001
	Oui	82	65	147	
Mode d’admission	Evacuées	202	76	278	0,0001
	Non évacuées	165	71	236	

Sur 531 naissances, il a été noté 10 morts fœtales intra-partum, soit un taux global de 18,8‰. Pour les 521 naissances vivantes, 91,5% avaient un score d’Apgar à une minute supérieur ou égal à 7. Il existait un lien statistiquement significatif entre le mode d’admission et l’état du nouveau-né ; en effet, le score d’Apgar inférieur à 7 était plus retrouvé lorsque la mère était évacuée. Nous avons enregistré 4 décès après quelques heures de vie suite à une asphyxie néonatale. Globalement, la mortalité périnatale était de 26,4‰ naissances vivantes et la mortalité néonatale précoce de 7,5‰ naissances vivantes. L’hématome rétroplacentaire et la rupture utérine étaient responsables respectivement de 50% et de 25% des morts fœtales in utero (MFIU). Il a été noté 2 décès maternels (0,4%). L’un à la suite d’une hémorragie grave du post-partum et l’autre dans un état de mal éclamptique.

## Discussion

La fréquence des accouchements sur utérus cicatriciel (9,6%) est sensiblement supérieure à ceux rapportés par la plupart des séries de la sous-région, mais inférieure à celle retrouvée par Deneux en France [8] (respectivement 40,8% et 11%). Cependant, des taux similaires au nôtre ont été retrouvés par Nayama au Niger [9] et Shi Wu Wen au Canada [10] qui sont respectivement de 9,7% et 9,8%. L’âge moyen des patientes était de 29,4 ans ; Baeta [11] et Cissé [7] retrouvaient la même moyenne d’âge que notre étude avec respectivement 29,5 et 29 ans. La parité moyenne dans notre étude était de 1,8%. Ce taux est inférieur à celui de Traoré [6] qui retrouvait une parité moyenne de 2,6. Les utérus unicicatriciels étaient les plus représentés avec une fréquence de 79,8%, les bi cicatriciels 17,9%, et les tricicatriciels 2,3%. Ces données sont en quasi-similitude avec celles de nombreux auteurs. Hamet [12] retrouvait respectivement 79,5%, 17,3%, et 3,2%. Cosson et coll [13], en France, répertoriaient respectivement 70,3%, 26,2%, et 3,5%. Même si cet ordre ne risque pas de s’inverser au fil des années, nous assisterons sûrement, avec l’inflation des césariennes, à une modification du rapport avec une hausse plus conséquente du taux des utérus multi-cicatriciels.

Dans notre série, plus de la moitié des patientes (52%) avaient un espace inter-génésique inférieur à 2 ans. Cet intervalle était inférieur à 1 an dans 29,5% des cas. Ce constat reflète parfaitement l’importance des besoins non couvert en contraception. En Afrique, où les moyens de surveillance (cardiotocographie) font souvent défaut, l’espace inter-génésique doit être prise en compte dans les critères de choix du mode d’accouchement. En effet, pour Cissé [7], une cicatrice datant de moins de 1 an est une indication de césarienne programmée. Shipp [14] retrouve un risque de rupture utérine trois fois plus important lorsque l’intervalle entre les deux accouchements est inférieur à 18 mois. En revanche, pour le Collège National des Gynécologues et Obstétriciens Français (CNGOF), un essai d’accouchement par voie basse peut être autorisé même en cas de délai inférieur à 6 mois si les conditions obstétricales sont favorables [4].

Dans notre série, environ 2/3 des patientes avaient accouché par césarienne (71,4%). Pour la plupart des auteurs, la césarienne était la voie d’accouchement la plus représentée. Ainsi, Cissé [7] et Traoré [6] retrouvaient respectivement 72,9% et 73,3% de césarienne. Cassignol [3] en France, et Nayama [9] au Niger retrouvaient respectivement 51,5% et 47,5% de césarienne. Le taux relativement élevé de césarienne dans notre série peut s’expliquer par le nombre important des indications permanentes de césarienne, à savoir les bassins rétrécis et les utérus multi-cicatriciels. A noter que l’utérus bi-cicatriciel est une indication formelle de césarienne à Dakar.

Les patientes évacuées représentaient 54,2% de notre série. Cependant, le taux élevé de patientes qui provenaient des structures sanitaires à vocation non chirurgicale (55,8%) atteste réellement du manque d’organisation du réseau périnatal dans nos districts sanitaires. Non seulement ces patientes sont suivies à 70% dans ces centres inadéquats à leur état, mais la référence ne s’est pas réalisée à temps, encore moins dans les conditions souhaitables (60,3%).

Dans notre série, 93 patientes (18,1% des cas) ont pu bénéficier d’une césarienne programmée. Ce taux est en deçà des ceux retrouvés par la majorité des auteurs. Ainsi Nayama [9] enregistrait 50,4% de césariennes programmées, et Traoré [6] 23,8%. Une fréquence de césarienne programmée inférieure à la nôtre a été retrouvée par Abassi et coll, qui rapportaient 13,8% [15]. En Europe [5, 16], la proportion de césarienne programmée sur utérus cicatriciel est passée de 34% à 48% entre 1999 et 2006. En réalité, si nous nous basons sur les indications de césarienne selon Boisselier, 229 patientes devraient systématiquement bénéficier d’une césarienne programmée, soit 44,5% des cas. Malheureusement, 67,7% de ces patientes étaient spontanément entrées en travail. Il faut noter par ailleurs que ces patientes étaient presque toutes suivies dans d’autres structures sanitaires périphériques.

Dans les césariennes programmées, les utérus multi-cicatriciels étaient au devant des indications avec 35,5%, suivis de très près, des bassins rétrécis (32,3%). Pour d’autres auteurs, le rétrécissement pelvien était le plus représenté. Abbassi [15] trouvait respectivement 46% des bassins rétrécis, et 13,8% des utérus multi-cicatriciels. Bien qu’à Dakar, les règles de l’obstétrique n’autorisent pas de double épreuve sur le bassin et sur l’utérus, 61,6% des patientes porteuses de l’utérus multi-cicatriciel avaient débuté le travail d’accouchement. Il ressort de ces résultats que des efforts restent à fournir dans la sensibilisation pour un suivi prénatal plus efficient et un respect rigoureux des normes et protocoles en santé de la reproduction.

Le taux létalité dans notre série est proche de celui retrouvé dans l’étude de Traoré [**6**] qui faisait état de 0,25%. Les auteurs sont unanimes sur le faible taux de mortalité, voire son absence, au décours des accouchements sur utérus cicatriciel [4, 9, 17], par crainte des risques maternels et périnatals. Concernant la morbidité maternelle, la fréquence des déhiscences de la cicatrice utérine était de 1% et celle de la rupture utérine était de 0,8%. Il faut cependant noter que les patientes qui présentaient une rupture utérine étaient toutes suivies dans des structures non chirurgicales et référées alors qu’elles étaient déjà en travail.

## Conclusion

La grossesse sur utérus cicatriciel est une grossesse à risque expliquant, en partie, l’augmentation de la pratique de la césarienne itérative. Cependant, Plus de la moitié des césariennes étaient faites en urgence. Ce constat doit nous pousser à réfléchir sur la nécessité d’une sensibilisation des prestataires sanitaires dans le but d’améliorer l’information des patientes.

### Etat des connaissances actuelles sur le sujet

- Le fait d’avoir une cicatrice utérine obstétricale met la patiente dans dans un groupe à risque de rupture utérine et d’hémorragie du post-partum;
- Les indications permanentes de césarienne doivent être connues au préalable par la patiente;
- La prise en charge des accouchements sur utérus cicatriciel n’est pas globalement codifiée.

### Contribution de notre étude à la connaissance

- La fréquence des césariennes augmente de manière considérable au Sénégal (9,6%);
- Du fait du manque d’information, le suivi prénatal des gestantes porteuses d’utérus cicatriciel se fait dans des structures non appropriées;
- Le transfert en urgence d’une parturiente porteuse d’utérus cicatriciel est un facteur de risque de césarienne.

## Conflits d’intérêts

Les auteurs ne déclarent aucun conflit d’intérêts.
